# Linkage to Care of People With Chronic Hepatitis B Virus and Hepatitis C Virus Infection Among Blood Donors: Experience From an Apex Treatment Centre Under National Viral Hepatitis Control Program, India

**DOI:** 10.1002/jgh3.70085

**Published:** 2024-12-22

**Authors:** Sk Mahiuddin Ahammed, Boby Maibam Singh, Swapan Saren, Pratik Dey, Raja Roy, Abhijit Chowdhury

**Affiliations:** ^1^ Department of Hepatology, School of Digestive and Liver Diseases Institute of Post Graduate Medical Education and Research Kolkata India; ^2^ Institute of Blood Transfusion Medicine, and Immunohematology Kolkata India; ^3^ Central Blood Bank Institute of Post Graduate Medical Education and Research Kolkata India; ^4^ Department of Microbiology Institute of Post Graduate Medical Education and Research Kolkata India

**Keywords:** cascade of care, hepatitis B virus, hepatitis C virus, linkage to care, National Viral Hepatitis Control Program

## Abstract

**Background and Objectives:**

Chronic viral hepatitis is a major public health challenge. The World Health Organization (WHO) and many national programs have set goals for elimination of viral hepatitis by 2030. Screening, Linkage to care (LTC), and access to treatment are very important steps to eliminate viral hepatitis. The study aimed to assess the cascade of chronic viral hepatitis care and the barrier of LTC in the National Viral Hepatitis Control Program (NVHCP).

**Methods:**

In this observational cross‐sectional study, healthy voluntary blood donors from two leading blood banks, who were HBsAg or anti‐HCV reactive, were advised to attend the clinic of NVHCP.

**Results:**

Among 116 569 healthy blood donors, prevalence of chronic HBV and HCV were 0.5% and 0.2% respectively. LTC was very poor. Only 27.7% HBsAg positive and 8.9% anti HCV positive patients attended NVHCP clinic. However, those who attended the clinic, 87.8% HBV and 100% HCV‐infected patients were retained. All HCV patients (*n* = 16) achieved SVR‐12. Among HBV‐infected patients, treatment eligibility was 21%.

**Conclusions:**

In this study, LTC was very poor. Only 27.7% of chronic HBV and 9% of HCV patients attended the NVHCP clinic. Immediate interventions are required to enhance LTC for asymptomatic patients with chronic viral hepatitis.

**Abbreviations**
ALT: alanine aminotransferase
APRI score: aspartate aminotransferase (AST)‐to‐platelet ratio index (APRI) score
AST: aspartate aminotransferase
BDL: below detection level
BMI: height and body mass index
CBC: complete blood count
DAA: direct acting‐antivirals
FIB4 score: fibrosis − 4 (FIB‐4) score
HBV: hepatitis B virus
HCV: hepatitis C virus
IDU: iv drug user
kPa: kilo‐Pascals
LFT: liver function
LS: liver stiffness
LTC: Linkage to care
NVHCP: National Viral Hepatitis Control Program
RFT: renal function tests
SVR12: sustained virological response 12
TE: transient elastography
WHO: World Health Organization

## 1 Introduction

Hepatitis B virus (HBV) and hepatitis C virus (HCV) causes considerable morbidity and mortality. According to the World Health Organization (WHO), an estimated 295.9 and 57.8 million people are living with chronic HBV and HCV infection respectively [1]. The WHO has set goals for eliminating viral hepatitis as a public health threat by 2030 [2, 3]. To meet WHO goals, we need to identify 90% of chronic viral hepatitis in community and we need to treat 80% HCV patients and 80% treatment eligible chronic HBV patients [3]. At present, only 9% of patients are aware of their virological status [4, 5]. Of this, only 10% of patients are linked to care and a minuscule fraction of affected individuals are currently receiving treatment, highlighting the urgent need to enhance access and ensure treatment for a larger proportion of those with chronic viral hepatitis [4, 5, 6]. Linkage to care (LTC) plays a crucial role in hepatitis management and act as a bridge between diagnosis and treatment [7]. A poor LTC is a major challenge for elimination of viral hepatitis [7]. Timely and efficient linkage will provide an opportunity to benefit from treatment at the earliest stage preventing disease progression and complications and reducing the disease burden in the community.

Recently, the Government of India has launched National Viral Hepatitis Control Programme (NVHCP), which aims to upscale screening, LTC and treatment in comprehensive manner to tackle viral hepatitis [8]. However, there is no study from India, which focuses on LTC among asymptomatic patients infected with viral hepatitis to NVHCP. The primary purpose of the present study is to evaluate and assess the LTC among healthy blood donors who have been found to be infected with chronic viral hepatitis.

### 1.1 Aims and Objectives

The aim of the study was to assess LTC among healthy blood donors infected with chronic viral hepatitis. This included (1) detecting the sero‐positivity of hepatitis B and C among healthy blood donors, (2) determining the number of seropositive persons linked to care, (3) assessing the number of persons retained in LTC, (4) identifying the number of persons initiated on therapy, (5) investigating barriers associated with poor LTC.

## 2 Methods

### 2.1 Study Design, Approvals and Participants

The study was conducted as an observational cross‐sectional study in the clinic of National Viral Hepatitis Control programme, School of digestive and liver disease, and IPGME&R, Kolkata, India. The study received ethical clearance from the IPGME&R Research Oversight Committee, vide Memo No. IPGME&R/IEC/2021/497. The study was conducted from April 2021 to September 2022. All patients were included after taking valid informed consent.

This study included voluntary blood donors through blood camp and replacement donor from two blood banks namely blood bank of IPGME&R, Kolkata and Central Blood Bank, Institute of Blood Transfusion Medicine, and Immunohematology, Manicktala, Kolkata, West Bengal, who were positive for HBsAg and Anti HCV antibody on blood screening. All the blood donors were between 18 and 65 years of age, physically active and healthy.

### 2.2 Screening for HBV and HCV

HBV infection is diagnosed upon detection of hepatitis B surface antigen (HBsAg) using HEPALISA KIT (Mitra &Co. Pvt. Ltd., India), an ELISA based test with sensitivity of 100% and specificity of 99.92% for detecting HBsAg.

Testing for HCV infection consist of detection of Anti HCV antibody using ELISA based HEPA‐SCAN KIT (Bhat Bio Tech India, Pvt. Ltd.), which is a third generation ELISA test with test sensitivity of 99% and specificity of 99.5%.

### 2.3 Defining Linkage to Care

LTC was defined as Individuals with positive for HBsAg or Anti HCV assay who attended the clinic of NVHCP, IPGME&R, Kolkata. Linkages to care was analyzed by Linked to care (attended 1 clinic appointment) and retained in care (attended > 1 clinic appointment) [9, 10]. Patients who tested positive for HBsAg or anti‐HCV were contacted and informed of their results by a counselor from the blood bank by using the contact details provided at the time of blood donation. This notification was done either directly (face‐to‐face) or by telephone. Subsequently, they were advised to attend the NVHCP clinic at our institute.

### 2.4 Data Collection, Assessment of Liver Disease and Initiation of Treatment

A complete data on socio‐demographic variables including age, sex, marital status, telephone/mobile numbers and address were recorded. Self‐reported risk factors for HBV and HCV transmission and history of addiction especially alcohol and smoking were recorded. Family history of viral hepatitis and hepatocellular carcinoma (HCC) or death due to liver disease was taken.

A comprehensive assessment of liver was done through physical examination, Liver function test (LFT), Complete blood count (CBC), Renal function test (RFT) and measurement of HBV DNA and HCV RNA quantitative assay, HBeAg/Anti‐HBeAb. HBV DNA and HCV RNA quantitative assay was done using PCR method [CobasAmpliPrep/CobasTaqMan 96 (ROCHE) Germany]. The lower limit of detection for HBV DNA and HCV RNA were 20 and 15 IU/mL respectively. HBeAg and HBeAb were tested using kit‐based ELISA method [DIA.PRO Diagnostic Bio probes Srl. (Italy)]. Abdominal ultrasonography with SPA Doppler was performed to look for the presence of cirrhosis and HCC. Non‐invasive tests (NITs) such as aspartate aminotransferase (AST)‐to‐platelet ratio index (APRI) score (AST [U/L]/upper limit of normal of AST)/(platelet count (10^9^ /L) × 100) [11], Fibrosis − 4 (FIB‐4) score (age [years] × AST [U/L])/(platelet count [10^9^ /L]) × √(ALT [U/L]) [12] were calculated. Liver stiffness (LSM) was measured with Transient Elastography (Fibroscan Expert 630, Echosens, France) An APRI score of more than 1.5 or FIB4 of more than 1.45 or LSM of ≥8kPa on transient elastography was considered as significant fibrosis (F2) whereas an APRI of more than 2 or FIB4 of more than 3.25 or LSM of ≥12.5kPa was considered as cirrhotic [13]. Upper GI endoscopy was done in cirrhotic patients to assess varices.

Patients were treated according to NVHCP guideline [8, 13].

### 2.5 Statistical Analysis

All data were entered into a Microsoft Excel Spreadsheet. Data analysis was carried out using appropriate statistical software using SPSS 28–2021 version. The results were expressed as percentage, median (Range) and mean+/− SD.

## 3 Results

**FIGURE 1:**
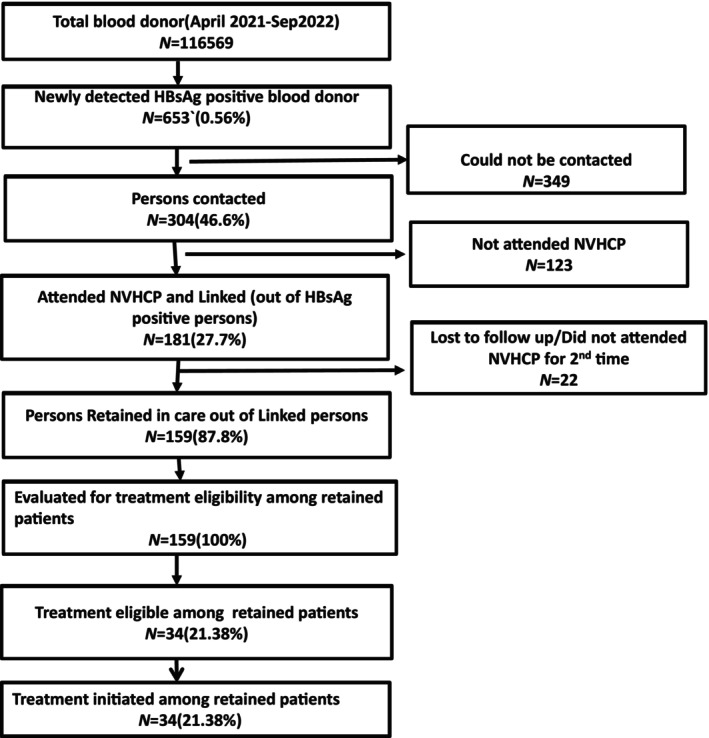
Consort diagram of HBV testing and linkage to care.

**FIGURE 2:**
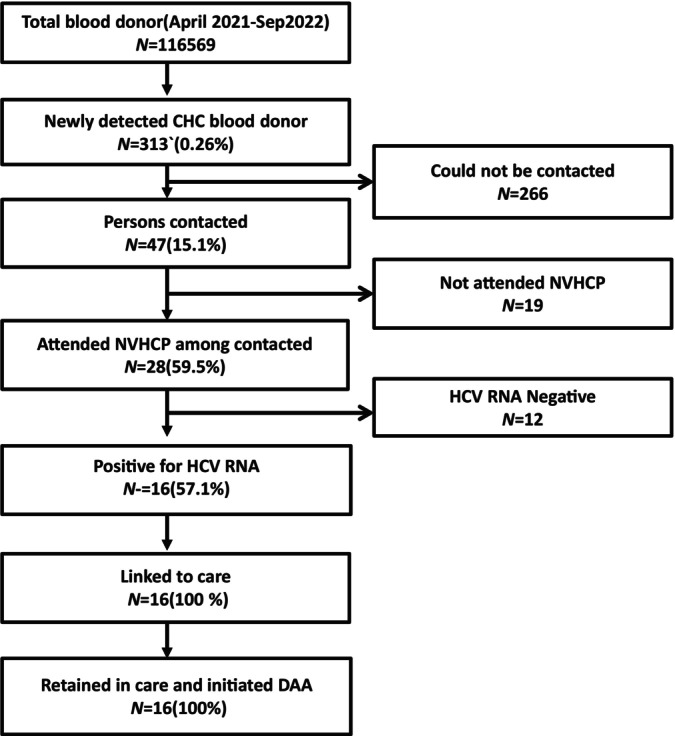
Consort diagram of HCV testing and linkage to care.

A total of 116 569 blood samples (42 075 from IPGMER Kolkata and 74 494 from IBTM &IH, Manicktala, and Kolkata) were screened for viral hepatitis. A total of 653 persons were found to be positive for HBsAg and 313 persons were found to be positive for Anti HCV antibody (Figures 1 and 2). Those patients, who were positive, were advised to attend NVHCP clinic of our institute.

### 3.1 HBV Infection and Linkage to Care

Out of 116 569, the HBsAg positivity rate was 0.56%. Of 653 persons positive for HBsAg, 304(46.5%) could be contacted (Figure 1). A total of 181 (27.7%) persons attended NVHCP clinic and were linked to hepatitis care (Figure 1).

### 3.2 Retained in Linkage

Of the 181 persons who attended the NVHCP clinic, 22 persons did not attend NVHCP for the second time (not retained) (Figure 1). The remaining 159 (87.8%) persons were classified as retained in linkage as they attended the NVHCP clinic at least two times. We evaluated these 159 persons for disease status and their detailed characteristics are given in Table 1. Of 159 patients, 13 8(86.8%) patients were male, 21 (13.2%) patients were female with Male: female ratio of 6.5:1. A family history of viral hepatitis is found in approximately 6.9% of individuals and family history of HCC found in 1.85% of individuals [table1]. Median age was 34 (18–56). The median ALT and AST were 33 U/L (12–221) and 34 U/L (16–126) respectively (Table 1).

**TABLE 1:** Base line characteristics of patients infected with HBV infection (*n*‐159).

Characteristics	Value
Age (year)^a^	34 (18–56)
Sex‐Male‐*n* (%)	138 (86.8%)
BMI (kg/m^2^)^b^	24.5 ± 3.7
Risk factors‐*n* (%)	
Blood transfusion	0 (0%)
Unprotected sex	4 (2.5%)
Tattoo	4 (2.5%)
Surgery	4 (2.5%)
IDU	0 (0%)
Family history‐*n* (%)	
HBV infection	11 (6.9%)
Hepatocellular carcinoma	3 (1.8%)
Death due to decompensated liver disease	3 (1.8%)
Addiction‐*n* (%)	
Alcohol Smoking Others	17 (10.7%) 16 (10.1%) 0 (0%)
Total Bilirubin^b^	0.81 ± 0.6
ALT (U/L) ^1^	33 (12–221)
AST (U/L) ^1^	34 (16–126)
Total protein (gm/dl)^b^	7.5 ± 0.52
Albumin (gm/dl)^b^	4.2 ± 0.35
APRI ^2^ < 1.5 1.5–2 > 2	0.54 ± 0.32 155 (97.5%) 4 (2.5%) Nil
FIB4^b^ < 1.45 (*n*) 1.45–3.25 (*n*) > 3.25 (*n*)	1.25 ± 0.75 146 (91.8%) 8 (5.0%) 5 (3.1%)
LSM (kPa)^b^ (*n* − 61) < 8 (*n*) 8–12.5 (*n*) > 12.5 (*n*)	5.7 ± 1.7 54 (88.5%) 6 (9.8%) 1 (1.7%)
HBV DNA (IU/ml) (*n*) > 20 000 2000–20 000 20–2000 < 20 (BDL)	32 (20%) 34 (21%) 81 (51%) 12 (8%)
HBeAg status (*n*) HBeAg negative HBeAg positive Not known	130 116 (89%) 14 (11%) 29
Cirrhosis (compensated) (*n*)	9 (6.7%)
No cirrhosis (*n*)	150 (94.3%)
Treatment eligible (*n*)	34 (21.3%)
Treatment initiated (*n*)	34 (21.3%)

Abbreviations: BDL‐below detection level; *n*‐no. of persons.

^a^ Median (range).

^b^ Mean + ‐SD.

In this study, 32 (20%) patients had high viremia (DNA > 20 000 IU/mL), 34 (21%) patients had moderate viremia (DNA 2000–20 000 IU/mL), and 81 patients (51%) had low viremia (DNA 20–2000 IU/mL) (Table 1). A total of 12 (8%) patients had an undetectable viral load (DNA < 20 IU/mL). HBeAg status was available for 130 patients, out of which 116 were HBeAg negative (89.3%) and 14 (10.7%) were HBeAg positive (Table 1).

Fibroscan data is available for 61 patients. Those who had normal APRI or FIB4 and USG they were not subjected to fibroscan. However, those who had APRI between 1.5 and 2 (*n* = 4) or FIB4 1.40–3.25 (*n* = 8) or family history of HCC (*n* = 3) and cirrhosis (*n* = 3) or altered echotexture of liver parenchyma without definite evidence of cirrhosis (*n* = 26) or significant alcohol intake (*n* = 17) were subjected to fibroscan by VCTE. Total 61 patients undergone fibroscan. A total of 54 patients (88.5%) had normal LSM (< 8 kPa) and 6 patients had LSM values between 8 and 12 kPa. Only 1 patient (1.7%) had an LSM value greater than 12.5 kPa (Table 1).

The mean APRI score and FIB4 score were 0.54 ± 0.32 and 1.25 ± 0.75 (Table 1). On the basis of APRI and FIB4 significant fibrosis was found in 2.5% and 5% respectively (Table 1). On the basis of non‐invasive tests, cirrhosis was diagnosed in 9 (6.7%) patients. Of these 9 patients, 5 patients were diagnosed on the basis of FIB‐4, 1 patient was diagnosed on the basis of LSM and 3 patients had endoscopic evidence of portal hypertension.

After evaluation, 34 persons (21.3%) were found to be eligible for treatment, as per NVHCP guidelines. Anti‐viral drug was initiated in all patients.

### 3.3 HCV Infection and Linkage to Care

Out of 116 569, the Anti‐HCV Ab positivity rate was 0.26% (Figure 2). Out of these 313 persons, 47 persons (15.0%) could be contacted (Figure 2). Out of 47 patients, 28(59.5%) patients attended NVHCP clinic. In 12 patients, HCV RNA was undetectable, so they were not linked. Thus 16 persons with positive viral load were finally linked and retained in linkage (Figure 2). The baseline characteristics of the sixteen patients are given in Table 2. The median age was 33.4 (20–57). All persons were male (100%). The median ALT was 59 U/L (20–164). The median HCV RNA was 23 094 IU/mL (344–9 899 494 IU/mL). The mean APRI score and FIB4 score were 0.76 ± 0.43 and 1.20 ± 0.52 (Table 2). On the basis of APRI and FIB4 none of the patient had cirrhosis. However one patient had radiological evidence cirrhosis with grade 1 ascites. All sixteen patients were treated with Sofosbuvir‐Daclatasvir combination for 12 weeks. All patients achieved SVR12 (Table 2).

**TABLE 2:** Base line characteristics of patients infected with HCV infection (*n*‐16).

Characteristics	Value
Age (year)^a^	33.4 (20–57)
Sex‐Male‐*n* (%)	16 (100%)
BMI (kg/m^2^)^b^	25.5 ± 3.4
Risk factors‐*n* (%)	
Blood transfusion	0 (0%)
Unprotected sex Tattoo Surgery IDU	2 (12.5%) 2(12.5%) 2 (12.5%) 4 (25%)
Family history‐*n* (%)	
Viral hepatitis	0 (0%)
Hepatocellular carcinoma	0 (0%)
Death due to liver disease	0 (0%)
Addiction‐*n* (%)	
Alcohol	3 (18.7%)
Smoking	3 (18.7%)
Others	4 (25%)
Total bilirubin^b^	0.80 ± 0.24
ALT (U/L)^a^	59 (20–164)
AST (U/L)^a^	50 (23–118)
Total protein (gm/dl)^b^	7.6 ± 0.63
Albumin (gm/dl)^b^	4.1 ± 0.37
APRI^b^	0.76 ± 0.43
< 1.5	15 (93.7%)
1.5–2	1 (6.3%)
> 2	0 (0%)
FIB4^b^	1.20 ± 0. 52
< 1.45 (*n*)	11 (71.7%)
1.45–3.25 (*n*)	5 (25.2%)
> 3.25 (*n*)	0 (0%)
HCV RNA (IU/ml) ^1^	23 094 (344–9 899 494)
Cirrhosis (*n*)	1 (6.3%)
No cirrhosis (*n*)	15 (93.7%)
Treatment initiated (*n*) SVR 12 achieved (*n*)	16 (100%) 16 (100%)

Abbreviation: *n*‐no. of persons.

^a^ Median (range).

^b^ Mean + SD.

### 3.4 Barrier to Linkage to Care

In order to identify factors contributing to successful linkage, several factors were assessed (Table 3). Detailed data are available for 177 patients who were successfully linked to care. Among them, there were 159 HBV‐infected patients who remained linked to care, 4 HBV patients who linked to care but were not retained, and 14 HCV patients who remained in care. Additionally, 78 HBV infected patients who were not successfully linked were willing to participate in telephonic interviews. Since the number of HCV patients is very small, we have not performed a statistical analysis to assess the LTC among HCV patients.

**TABLE 3:** Factors associated with linkage to care in HBV infected patients.

	Linked to care (*n* = 163)	Not linked (*n* = 78)	*p*
Age (year)^a^	35 (18–58)	33.5 (21–56)	0.80
Sex			0.30
Male *n* (%)	136 (83.4)	73 (93.6)	
Female *n* (%)	27 (16.6)	5 (6.4)	
Social stigmata			0.18
Faced *n* (%)	9 (5.5)	8 (10.3)	
Not faced *n* (%)	154 (94.5)	70 (89.7)	
Prior knowledge of NVHCP			0.95
Present *n* (%)	4 (2.5)	2 (2.6)	
Absent *n* (%)	159 (97.5)	76 (97.4)	
Properly linkage			< 0.001
Yes *n* (%)	163 (100)	58 (74.3)	
No *n* (%)	0 (0)	20 (25.6)	
Socio‐economic status^b^			0.12
Upper class *n* (%)	0 (0)	0 (0)	
Upper middle *n* (%)	5 (3.1)	0 (0)	
Lower middle *n* (%)	27 (16.6)	10 (12.8)	
Upper lower *n* (%)	106 (65)	52 (66.7)	
Lower class *n* (%)	25 (15.3)	16 (20.5)	
Rural versus urban			0.71
Rural *n* (%)	90 (55.2)	45 (57.7)	
Urban *n* (%)	73 (44.8)	33 (42.3)	
Education			0.11
Illiterate *n* (%)	31 (19)	20 (25.6)	
Primary education *n* (%)	30 (18.4)	15 (19.2)	
Middle *n* (%)	56 (34.4)	29 (37.2)	
High school *n* (%)	17 (10.4)	4 (5.1)	
Intermediate *n* (%)	3 (1.8)	0 (0)	
Graduate *n* (%)	26 (16)	10 (12.8)	
Failure to attend NVHCP			
Asymptomatic *n* (%)	0	44 (56.4)	
Loss of daily wages *n* (%)	0	19 (24.4)	
Prefer to treat in private hospital *n* (%)	0	15 (19.2)	

^a^ Median (ranges).

^b^ Kuppuswamy scale according to family income (INR) based on year 2022.

Prior knowledge of NVHCP was poor in both groups. Only 4 patients in the linked‐to‐care group had knowledge; 3 of them had a family history of HBV and were undergoing treatment at NVHCP. One was a healthcare worker who was familiar with NVHCP. In the not‐linked‐to‐care group, 2 patients knew about the NVHCP program (Table 3). One patient had a family history of HBV and was advised to attend NVHCP care for viral load estimation, while another patient learned about NVHCP through public media. This indicates an overall poor awareness of NVHCP among the general population. The study provided data to suggest a significant association between receiving proper instructions regarding the name of the centre, date of outpatient department (OPD), and OPD schedule, and the likelihood of seeking treatment (*p* = 0.001) [Table 3]. Specifically, individuals who were given these instructions were more inclined to seek treatment. In the linked‐to‐care group, where patients successfully sought treatment, it's noteworthy that all individuals received proper instructions. However, in the unsuccessful group, comprising those who did not seek treatment, only 74% (*n* = 79) received proper instructions, while the remaining did not (Table 3).

The disparity in beliefs between the unsuccessful group and the linked‐to‐care group is striking. In the unsuccessful group, a significant proportion (56.4%) of patients held the belief that they were asymptomatic and either did not have the disease or were not at risk of developing it in the future. Conversely, in the linked‐to‐care group, all individuals acknowledged that they had liver disease or were at risk of developing it in the future, hence recognizing the necessity for treatment (Table 3). The fear of losing daily wages was high among the failure‐to‐link to care group, whereas none of the successfully linked to care group expressed fear of losing daily wages (Table 3). Other demographic and socio‐economic factors were not significantly associated with LTC (Table 3).

## 4 Discussion

LTC refers to the process of connecting individuals who have been recently diagnosed with chronic viral hepatitis to treatment centre [7, 9, 10]. Timely LTC ensures that individuals receive antiviral therapy, monitoring, and counseling, to prevent disease progression and transmission. Government agencies and National Program play significant roles in developing and implementing strategies to improve LTC for HBV and HCV. There are only few national program which aim screening, LTC and treatment. Govt. of India recently introduced NVHCP with objective to eliminate viral hepatitis by 2030 [13]. However, there is no data from India which evaluated linkage to NVHCP. So, we did this observational cross‐sectional study to understand linkage to national program and barrier to linkage. This study may strengthen NVHCP and may foster elimination of viral hepatitis.

In this study a total of 116 569 blood samples were screened. The prevalence of HBV positivity among blood donors was 0.56% which was lower than some other studies done in India with a prevalence ranging from 0.66% to 2.63% [14, 15, 16]. The prevalence of Anti HCV positivity among the blood donors was 0.26%.

In this study a low rate of linkage was seen among HBV patients. We found that 181 persons (27.7%) of all HBsAg positive persons (*n* = 653 persons), attended NVHCP clinic. Among HCV positive persons we also found a low rate of linkage. Only 28 patients attended clinic of NVHCP. Gilberto Ramirez et al. in their study also showed a linkage rate of 46% for HBV and 44% for HCV among those who were positive for HBsAg and HCV RNA respectively which was higher than our study finding [17]. Another study also demonstrated that even with implementation of a structured intervention for high‐risk patients, only 52% of patients newly diagnosed with HCV infection attended specialist appointments [18]. We found a relatively high rate of retained in care in both HBV and HCV with a rate of 87.8% and 100% respectively. Similarly in a study by Hannah Evans et al. 93% of HBV and 78% of HCV patients were retained in care [10]. One elegant study by Dhiman et al. demonstrated that the decentralized “Punjab model of hepatitis C care” is safe and very effective [19]. However, this study does not address LTC or the factors associated with LTC.

Thus, we found that major problem in the LTC continuum was in the initial part of linking of patients to the linkage system.

In our study 21.3% patients were eligible for HBV treatment. All the 16 retained viremia patients were treated with DAA and achieved SVR12.

In this study, several factors may have acted as barriers to achieving a satisfactory linkage rate to care. Proper LTC was identified as a major obstacle. Few simple interventions could potentially improve the LTC process. Firstly, providing comprehensive information about treatment centres, including their names, OPD dates and times, and the name of a contact person, may enhance LTC. This information dissemination could be facilitated through repeated telephone calls, text messages, or distributing leaflets containing all necessary details. Another major obstacle was the lack of awareness regarding the disease and the outcomes of leaving it untreated. This underscores a critical barrier to seeking treatment among individuals who perceive themselves as asymptomatic. Such perceptions can lead to underestimating the seriousness of the condition and delaying medical care‐seeking. Sensitization efforts aimed at addressing the lack of knowledge about the disease and its treatment may be beneficial, as the majority of patients lacked proper understanding of viral hepatitis. In the context of the unsuccessful group mentioned, where individuals did not seek treatment, it is plausible that the fear of losing daily wages played a crucial role in their decision‐making process. Addressing economic barriers to healthcare, such as providing financial assistance can help mitigate the impact of wage loss on healthcare‐seeking behavior.

Further studies concentrating on the various barriers to linkage may be required to better understand the root causes for addressing these issues at the community level for further strengthening the existing programmes.

## 5 Limitations

There are few limitations of this study. First, the study included blood donors from two blood banks only. Inclusion of other blood bank could have changed the linkage rate. Second, though NVHCP is a decentralized health care system, irrespective of the domicile of the patients, they were advised to attend the NVHCP clinic of our institute only. It may be inconvenient for the patients. Third, the government health care system is not always seamless; few patients might have attended private health care system, details of which are unknown. Fourth, patients were contacted via telephone, which comes with several limitations. Fifth, assessing the influence of the health care facility on LTC is crucial. Factors such as accessibility, quality of care, availability of resources, and staff attitudes can significantly impact an individual's decision and ability to engage in care after diagnosis. However, this aspect was not assessed.

## 6 Conclusion

In order to eliminate chronic viral hepatitis as a public health threat, it is crucial to ensure adequate LTC for individuals who are infected with chronic viral hepatitis. Though National Hepatitis Control Program provides complementary diagnostic support and antivirals, 72.3% HBV and 91% HCV infected patients did not attend treatment centre. However, those who attended NVHCP, most of them are retained in care. Inadequate LTC is a major obstacle to eliminate viral hepatitis from India. New strategies and interventions are urgently needed to improve LTC among asymptomatic patients infected with chronic viral hepatitis.

## Conflicts of Interest

The authors declare no conflicts of interest.
